# Three-dimensional spheroid culture targeting versatile tissue bioassays using a PDMS-based hanging drop array

**DOI:** 10.1038/s41598-017-04718-1

**Published:** 2017-06-29

**Authors:** Ching-Te Kuo, Jong-Yueh Wang, Yu-Fen Lin, Andrew M. Wo, Benjamin P. C. Chen, Hsinyu Lee

**Affiliations:** 1 Division of Molecular Radiation Biology, Department of Radiation Oncology, University of Texas Southwestern Medical Center, Dallas, TX USA; 2 Department of Life Science, National Taiwan University, Taipei, Taiwan, ROC; 3 Institute of Applied Mechanics, National Taiwan University, Taipei, Taiwan, ROC

## Abstract

Biomaterial-based tissue culture platforms have emerged as useful tools to mimic *in vivo* physiological microenvironments in experimental cell biology and clinical studies. We describe herein a three-dimensional (3D) tissue culture platform using a polydimethylsiloxane (PDMS)-based hanging drop array (PDMS-HDA) methodology. Multicellular spheroids can be achieved within 24 h and further boosted by incorporating collagen fibrils in PDMS-HDA. In addition, the spheroids generated from different human tumor cells exhibited distinct sensitivities toward drug chemotherapeutic agents and radiation as compared with two-dimensional (2D) cultures that often lack *in vivo*-like biological insights. We also demonstrated that multicellular spheroids may enable key hallmarks of tissue-based bioassays, including drug screening, tumor dissemination, cell co-culture, and tumor invasion. Taken together, these results offer new opportunities not only to achieve the active control of 3D multicellular spheroids on demand, but also to establish a rapid and cost-effective platform to study anti-cancer therapeutics and tumor microenvironments.

## Introduction

Three-dimensional (3D) tissue cultures show distinct characteristics in terms of cellular heterogeneity/plasticity/morphology, mass transport, and complex cell-matrix or cell-cell interactions as compared with conventional 2D cell cultures^1–3^. In addition, cancer cells cultured in 3D mimic the *in vivo* microenvironments more closely. In contrast, the malignant phenotypes and the mechanotransduction between extracellular matrix (ECM) and cells dramatically diminish in 2D^4–7^. Because of their ability to mimic human physiological conditions and integrating with high-throughput and high-content techniques, biomaterial-based tissue culture platforms are ideal tools to address these critical issues. These platforms have emerged as useful tools to explore fundamental aspects of cell biology, tissue engineering, and drug development, with broader impacts in medical applications^8, 9^.

Different techniques have been tested for 3D tissue culture platforms including organic^10, 11^ or inorganic matrix^12, 13^ coating on plastic substrates, paper-supported scaffolds^2, 14^, magnetic levitation of cells^15^, and hanging drops^16^. Although these platforms provide a desirable environment for 3D culture and testing, challenges still remain for their use. For example, alternative approaches are needed to cooperate with automatic liquid handing for large bioassays. Moreover, some techniques typically require a large amount of cells to perform 3D cell cultures, resulting in cell waste and restricting potential applications, e.g. circulating tumor cells (CTCs)^17^ and primary tumor samples^18^. Other challenges include the use of agarose matrices or engineered nanoparticles for 3D cultures, which may affect the cells both biochemically and physiologically. Despite the progress on modeling the tumor microenvironment *in vitro* ^19–21^, experiments on cell motility, dissemination, cell co-culture, and matrix invasion are still difficult to conduct.

Herein we present a polydimethylsiloxane (PDMS)-based hanging drop array (PDMS-HDA) platform that produces deterministic 3D spheroid cultures in a high-throughput manner. The forming efficiency of the cellular spheroids was improved after interacting with collagen fibrils at a rate of 24 h or less. We also showed that a small number of cells is sufficient to generate 3D spheroids (around 200 cells per spheroid). Furthermore, we demonstrated that this platform can be used to determine the phenotypic characteristics of the crucial cancer hallmarks mentioned earlier. This platform may be used as a cost-effective and purpose-tailored toolkit to study tumor cell biology.

## Results and Discussion

### The combination of PDMS coating and collagen fibrils promotes tumor spheroid growth in hanging drops

**Figure 1:**
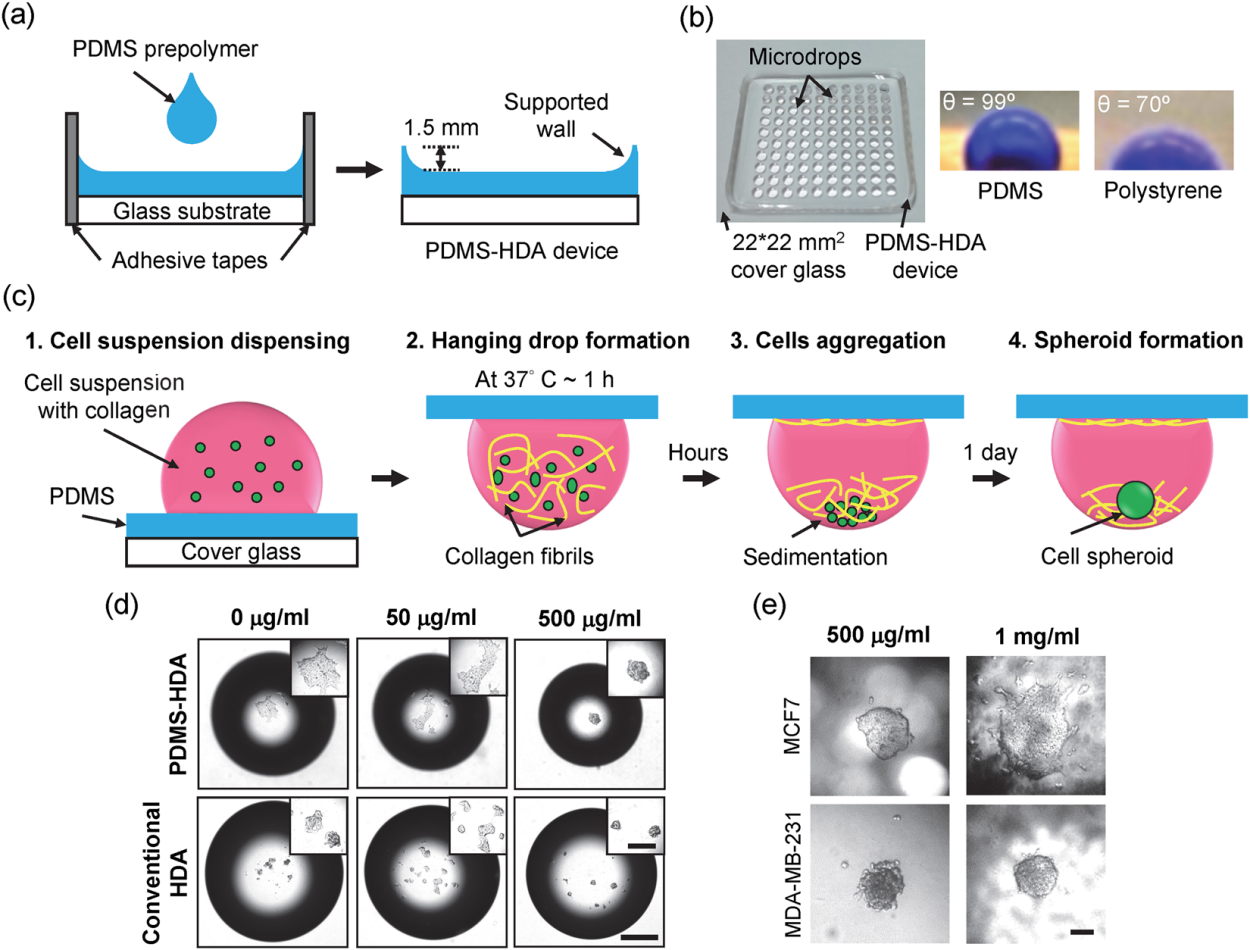
Three-dimensional spheroid culture using a PDMS-based hanging drop array (PDMS-HDA). (**a**) Fabrication of the PDMS-HDA device. (**b**) The left pictures show the fabricated PDMS-HDA device consisting of a 10*10 array of 1 μl drops dispensed by a liquid handing machine. The two micrographs on the right show the DI water drops with blue dyes on PDMS and polystyrene surfaces. (**c**) Procedure of 3D cell culture by the PDMS-HDA device. (**d**) Comparison of 3D MCF7 spheroids between the PDMS-HDA device and conventional HDA at different concentrations of collagen-I supplemented in medium at 24 h. The initial cell numbers per drop were 450. Scale bar, 500 μm; insert bar, 200 μm. (**e**) Comparison of MCF7 and MDA-MB-231 spheroids performed in 500 μg/ml and 1 mg/ml collagen-contained medium drops, respectively. Scale bar, 100 μm.

We have employed the commonly-used hanging drop array (HDA) technique^16, 22, 23^ to develop miniature tumor spheroids in culture. The improved methodology relied on the interaction between PDMS surfaces and collagen fibrils Fig. 1(a–c). PDMS was found to be nontoxic and biocompatible in many cellular studies^24^. Because of its hydrophobic nature and a contact angle of 99°, PDMS can hold micro-scaled drops dispensed by a liquid handing machine Fig. 1(b). To demonstrate the feasibility of using both PDMS surfaces and collagen fibrils to improve spheroid culture, different collagen mixtures were tested using MCF7 cells for spheroid growth rate and cell viability Figs 1(d,e), and S1, Supplementary Information. As shown in Fig. 1(d), spheroid formation from our PDMS-HDA was significantly improved compared with the conventional HDA approach^25^. Our results indicate that supplementing the medium mixture with 500 μg/ml collagen is the optimal condition to induce the growth of a single and compact spheroid of MCF7 and MDA-MB-231 cells hung by the PDMS surface Fig. (1e). MCF7 cells cultured in 500 μg/ml collagen-I mixture formed unique spheroids resembling those found in mammospheres^4^ and could sustain almost 100% viability for a long-term observation Figure S1(a) and (b).

To the best of our knowledge, the hanging approach is rarely used to achieve tumor spheroids using only a handful of cells in microarrays and to yield a critical volume of 1 μl or less. Therefore, this type of system is urgently needed to harvest clinical samples with limited cell counts. Following the typical procedure of conventional hanging drop culture^25^, MCF7 cells in a 1-μl medium drop were added to adhere onto the flipped surfaces of either PDMS or polystyrene after a 1-day culture Fig. 1(d): the results of without collagen, rather than forming cellular aggregates or spheroids by 10-μl drops^22, 25^. These findings suggest that the interfacial forces between the surfaces and the cell drops with a critical volume could attenuate the efficiency of cell aggregation, questioning the feasibility of the conventional hanging drop technique for 3D cell microarrays.

Unique and compact MCF7 and MDA-MB-231 spheroids were formed by the hanging cell drops supplemented with a critical 500 μg/ml collagen-I mixture on PDMS surfaces, whereas multiple cell aggregates were found by the hanging cells on polystyrene surfaces. In addition, single MDA-MB-231 spheroids were observed in a collagen-mixed drop with a higher concentration (i.e. 1 mg/ml), whereas MCF7 cells presented a loose 3D morphology Fig. 1(e). This morphology is consistent with a previous report showing that collagen-I can promote aggressive or metastatic breast cancers (e.g. MDA-MB-231) more than non-metastatic cancers (e.g. MCF7)^26^. Furthermore, the diameter of each dispensed drop (1 μl) on PDMS was 1.4 mm as compared with that of 1.8 mm on polystyrene, thereby generating a more concentrated cell-collagen mixture to form cell spheroids due to the hydrophobic nature of PDMS. These results demonstrate that the 3D culture approach by PDMS-HDA may achieve the *in vitro* modeling of tumor spheroids with a critical volume.

### Three-dimensional cell culture reveals diverse characteristics of drug sensitivity compared with 2D culture

**Figure 2:**
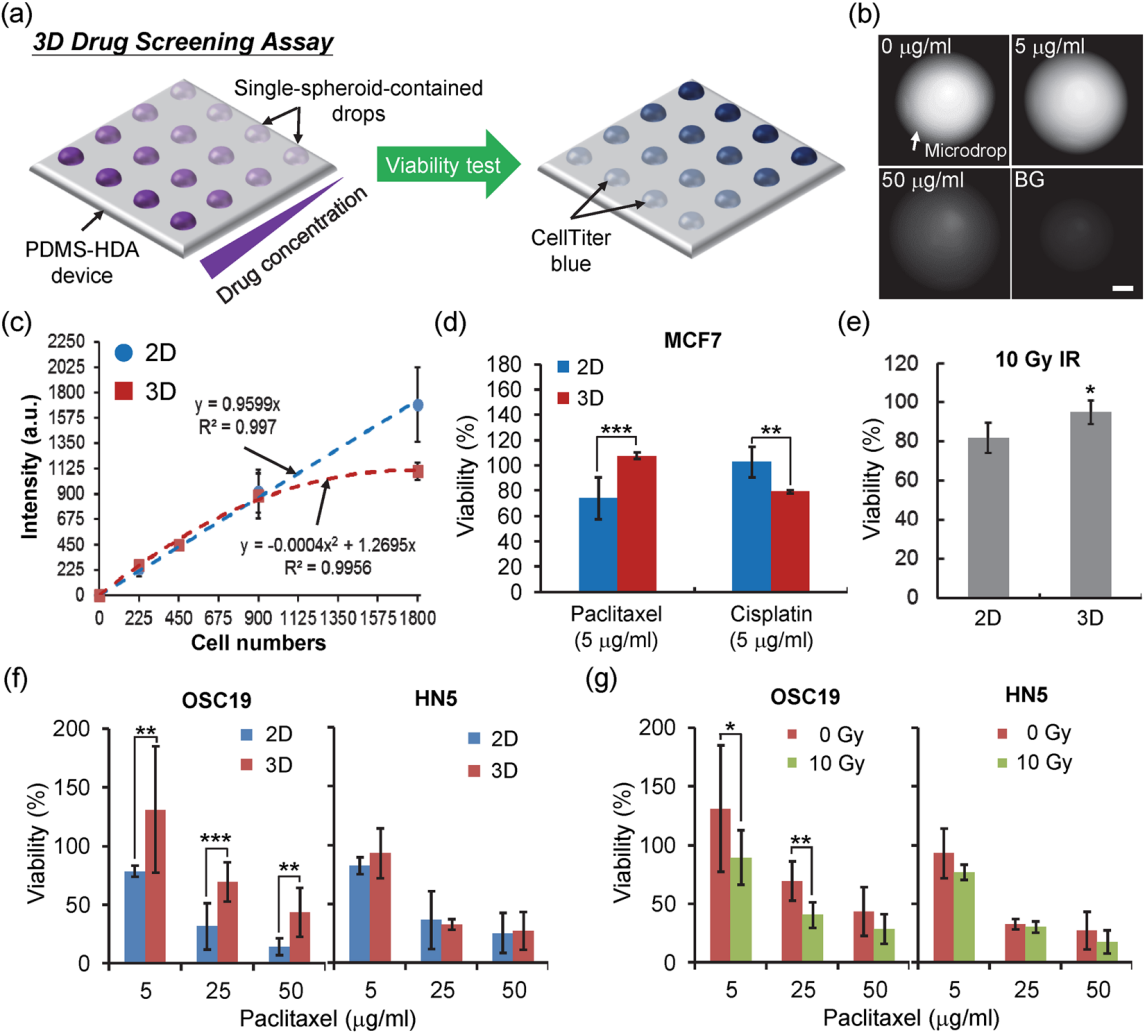
Three-dimensional cell culture by PDMS-HDA reveals diverse drug sensitivity characteristics compared with 2D culture. (**a**) Procedure of drug screening by PDMS-HDA. (**b**) Fluorescent images showing the single MCF7 spheroid-containing drops dispensed with Celltiter-Blue after incubation for 4.5 h. The cellular spheroids were treated with paclitaxel at different concentrations for 2 days before examining cellular viability. BG represents the drop that contained no cells. Scale bar, 500 μm. (**c**) Relationship between the initial cell number per drop and the resulting fluorescent intensity of the CellTiter-Blue. Each data represents the mean ± SEM (n = 3~4). a.u., arbitrary unit. (**d**) Drug responses of 2D- and 3D-cultured MCF7 cells treated with paclitaxel or cisplatin (5 μg/ml) for 48 h. The data are presented as mean ± SEM from 2 independent experiments (n = 8~10) of paclitaxel treatment and from 3 experiments (n = 11~12) of cisplatin treatment. (**e**) Irradiation sensitivities of MCF7 cells cultured in either 2D or 3D over 2 days after γ-ray treatment. The data are presented as mean ± SEM from 2 independent experiments (n = 8~9). (**f**) and (**g**) Dose responses of OSC19 and HN5 cells treated with paclitaxel at different concentrations (cells either cultured in 2D or 3D) and with the combined treatment of paclitaxel and irradiation (cells cultured in 3D) for 2 days. Each bar represents the mean ± SEM from 3 independent experiments (n = 9~15). *p < 0.05, **p < 0.01, and ***p < 0.001 indicate statistical significance.

To assess the chemosensitivity of monolayer cells and cells under spheroid culture using the PDMS-HDA device, we investigated paclitaxel and cisplatin, two commonly used chemotherapeutic drugs. The drugs were subjected to 2D and 3D cultures for 48 h at different concentrations up to 50 μg/ml Fig. 2(a). The cellular viability was measured using a CellTiter-Blue assay that provides good correlations between the detected fluorescence and the cell numbers for each spheroid Fig. 2(b) and (c), as 1^st^-order and 2^nd^-order fitting equations for 2D and 3D conditions, respectively. No significant difference in dose response was observed between the conventional 96-well plate 2D and the PDMS-HDA 3D cultures, suggesting that the device may be compatible for drug screening (Figure S2, Supplementary Information). The viability of untreated MCF7 spheroids over a total 3-day culture was nearly 100% (Figure S3, Supplementary Information). Our analyses revealed that, under drug treatment (spheroid growth for 2 days and then drug treatment for 24 h), 3D-cultured MCF7 cells were more resistant to paclitaxel but more susceptible to cisplatin than cells cultured in 2D Fig. 2(d), see also Supplementary Figure S4(a) and (b). Furthermore, 3D MCF7 spheroids were more resistant to ionizing radiation (IR) than monolayer cell cultures Fig. 2(e), similarly to a previous study using the clonogenic survival assay^14^. Likewise, MDA-MB-231 spheroids were resistant to both paclitaxel and cisplatin Figure S4(c) and (d), Supplementary Information. Two-dimensional and 3D cultures were also compared using head and neck squamous cell carcinoma (HNSCC) cells Fig. 2(f,g), and Supplementary Figure S4(e) and (f). The difference in dose response between HNSCC OSC19 and HN5 cells indicated that drug sensitivity depends on the cell type in either 2D or 3D, although 3D cell cultures reflected the *in vivo*-like microenvironment more effectively.

Tumor spheroid culture is generally more resistant to anti-cancer drugs than 2D culture. For example, MCF7 spheroid-enriched cells are resistant to cisplatin because they acquire cancer stem cell properties^27^. However, this result is in contrast with our data obtained with cisplatin Fig. 2(d). This discrepancy may be explained by the cells being cultured under stem cell-enriched conditions in the previous study^27^, which is in contrast with our method based on standard medium containing no additional growth factor supplements to enforce cell reprogramming. Our previous study demonstrated that MCF7 spheroids of first generation are sensitive to cisplatin added to the parental cells at a dose range of 0~50 μg/ml^28^, which is consistent with the data presented in this work Fig. 2(d). We reported that a downregulation of hypoxia-inducible factor 1 alpha (HIF-1α) in MCF7 spheroids contributes to cisplatin sensitivity^28^. HIF-1α is known to positively regulate expression of DNA repair molecule Xeroderma pigmentosum complementation group A (XPA) and leads to cisplatin resistance in lung cancer^29^. The distinct responses between 2D and 3D cultures to the therapeutic treatments highlight the urgent need of 3D culture platforms in drug screening and development.

### Tumor spheroid dissemination assay

**Figure 3:**
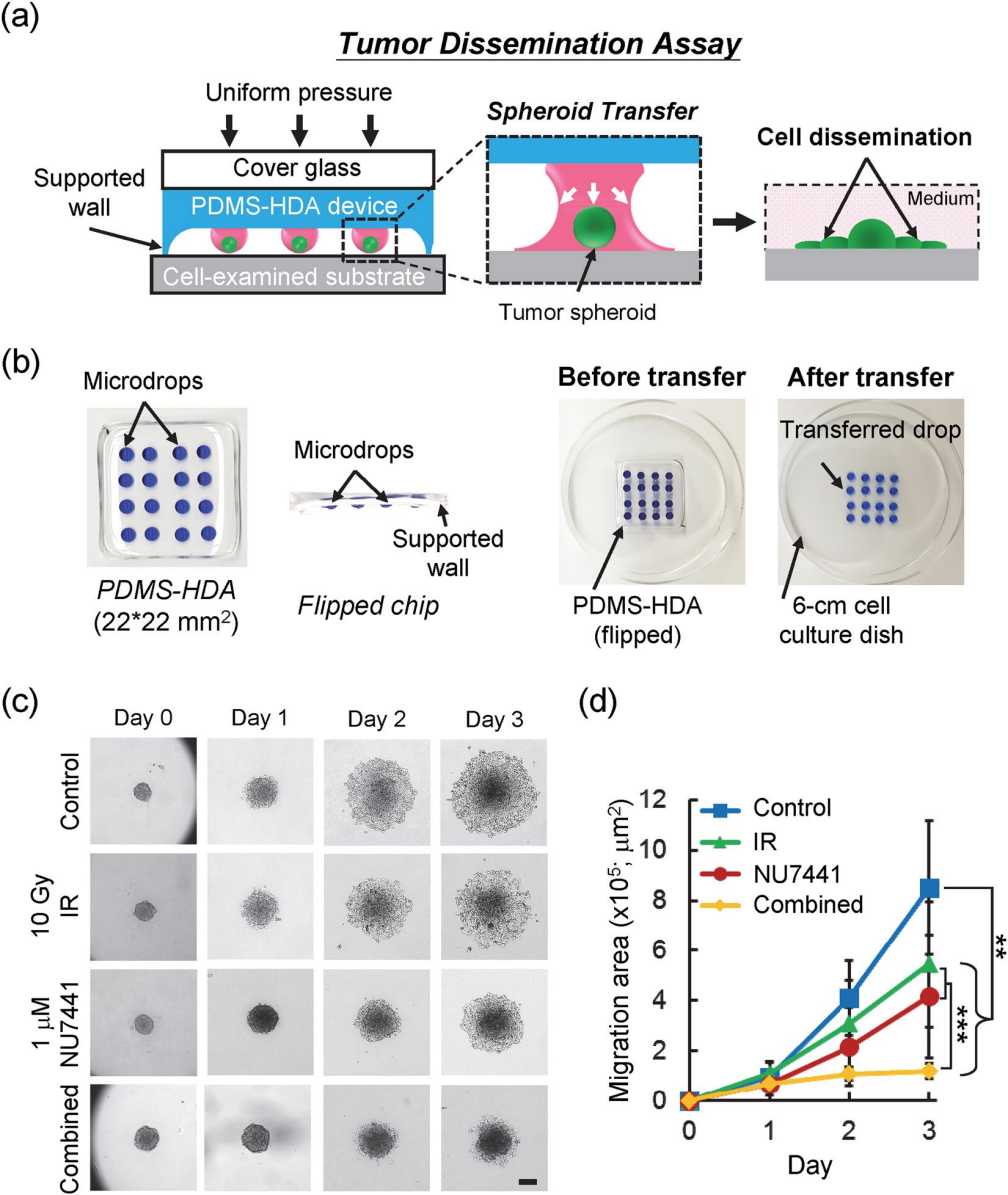
Tumor spheroid dissemination assay. (**a**) Design concept of the tumor dissemination assay by PDMS-HDA. (**b**) Example of *in-situ* transfer of the cell spheroid-containing drop array (4*4 array highlighted by blue dyes; 11 μl per drop) from the device to the appropriate cell-examined substrate (e.g. cell culture dish). (**c**) Time-sequenced images showing the dissemination of HCT116 spheroids over a 3-day culture, in which cells were treated with irradiation, NU7441, or combined treatment from day 0. Scale bar, 200 μm. (**d**) Spheroid migration area correspondingly measured from day 0 to 3. Each data represents the mean ± SEM from 2 independent experiments (n = 17~29; **p < 0.01, ***p < 0.001).

Tumor cell dissemination occurs during tumor development and is the hallmark of cancer metastasis. The ability of tumor spheroids to disseminate can be tested by placing them onto cell-examined surfaces to allow cells migrate out from those spheroids. The process mimics tumor cells migration from a small tumor cluster or micrometastasis through engagements with host stromal matrix proteins^21, 30^. Despite its usefulness, the assay presents several obstacles; for example, the transfer of numerous cell spheroids to a cell-examined substrate is time-consuming and laborious. While some modified receiver plates have improved the transfer step^23, 31^, we demonstrated that the *in situ* dissemination assay can be easily executed using the PDMS-HDA device Fig. 3(a) and (b). The average volume of the shifted drops was around 5 μl, indicating that an approximate volume loss of 55% (for a total volume of 11 μl) occurs during the transfer step. The efficiency of transferring the spheroids was as high as 96% in 2 independent experiments (n = 8) Fig. 3(c). Our proof-of-concept result indicated that spheroid dissemination can be achieved for continuous observation. Our further analyses demonstrated that HCT116 spheroid dissemination *in situ* was attenuated by treating the cells with 10 Gy IR and DNA-PK kinase inhibitor NU7441 Fig. 3(c) and (d). Furthermore, two distinctly disseminated patterns were observed in MCF7 and MDA-MB-231 breast cancer cells with collective and individual migration, respectively (Supplementary Figure S5). These findings indicate that cancer dissemination strongly depends on the migratory cells produced during metastasis^32, 33^.

### Three-dimensional tumor spheroids co-culture assay

**Figure 4:**
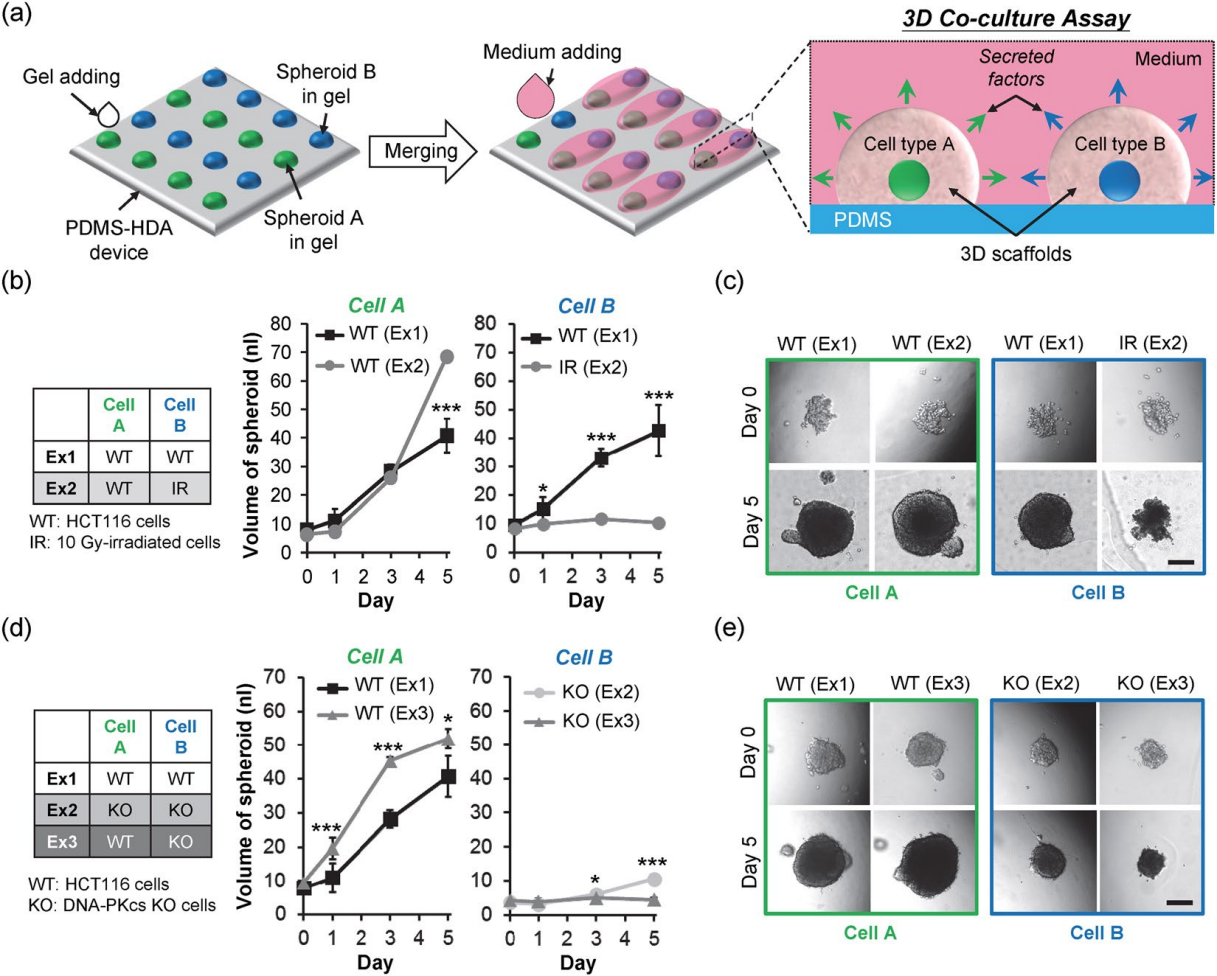
Three-dimensional cell co-culture assay. (**a**) Design concept of the 3D co-culture assay by PDMS-HDA. (**b**) Results obtained with the volumes of cellular spheroids evaluated over a 5-day culture: culture of two compartmentalized wild-type (WT) HCT116 spheroids (Ex1) and co-culture of one WT spheroid and one irradiated spheroid (Ex2). (**c**) Corresponding morphologies of each spheroid at days 0 and 5. (**d**) Results obtained after comparing HCT116 spheroids from the co-culture of two compartmentalized WT spheroids (Ex1), two separate DNA-PKcs −/− (KO) spheroids (Ex2), and two separate WT and KO spheroids (Ex3) over 5 days. (**e**) Corresponding morphologies of each spheroid at days 0 and 5. Each data represents the mean ± SEM from 2~3 independent experiments (n = 4~8; *p < 0.05, ***p < 0.001). Scale bars, 200 μm.

Previous studies reported that cell death or senescence caused by persistent DNA damage after irradiation triggers inflammatory cytokine and signaling molecules released from injured cells to communicate with their surrounding tissue and to stimulate cell repopulation during tissue recovery^34, 35^. Since those results were obtained from *in vivo* assays, an accessible *in vitro* assay platform will facilitate mechanistic investigations and high-throughput drug analyses. For proof-of-concept, we have modified our PDMS-HDA platform to co-culture two different tumor spheroids Fig. 4(a). Two spheroid co-culture models were studied for cell communication and phenotypic characteristics by the PDMS-HDA device. First, the volume of untreated HCT116 spheroids increased after 5 days of co-culture with injured HCT116 spheroids that were previously irradiated with 10 Gy, as compared with HCT116 spheroids co-cultured with untreated spheroids of the same cell type Fig. 4(b) and (c). In addition, the irradiated spheroids not only underwent growth arrest but also showed a loose 3D cell aggregate over the 5-day co-culture. Our results are consistent with previous studies reporting that cells injured by irradiation could promote the proliferation of nearby undamaged cells^34, 35^. Second, HCT116 spheroids were tested in co-culture with HCT116-derived DNA-PKcs (the catalytic subunit of the DNA-dependent protein kinase) knockout (KO) cells Fig. 4(d) and (e). A previous study reported that inhibition of DNA-PKcs could enrich the senescent cell population in human cancer^36^. Our results showed that the growth rate of HCT116 spheroids was significantly higher when co-cultured with the KO spheroids over 5 days than that co-cultured with the same HCT116 spheroids. This finding is also in agreement with our observation on growth stimulation with cells treated with irradiation. We postulated that the KO spheroids could promote the growth of nearby WT cells due to stimuli from the senescence-associated secretory phenotype (SASP) factors^34^. Therefore, the PDMS-HDA platform could be applied to a wide range of cell co-culture studies, including cell-cell communication and secretory factor analyses.

### Three-dimensional tumor spheroid invasion assay

**Figure 5:**
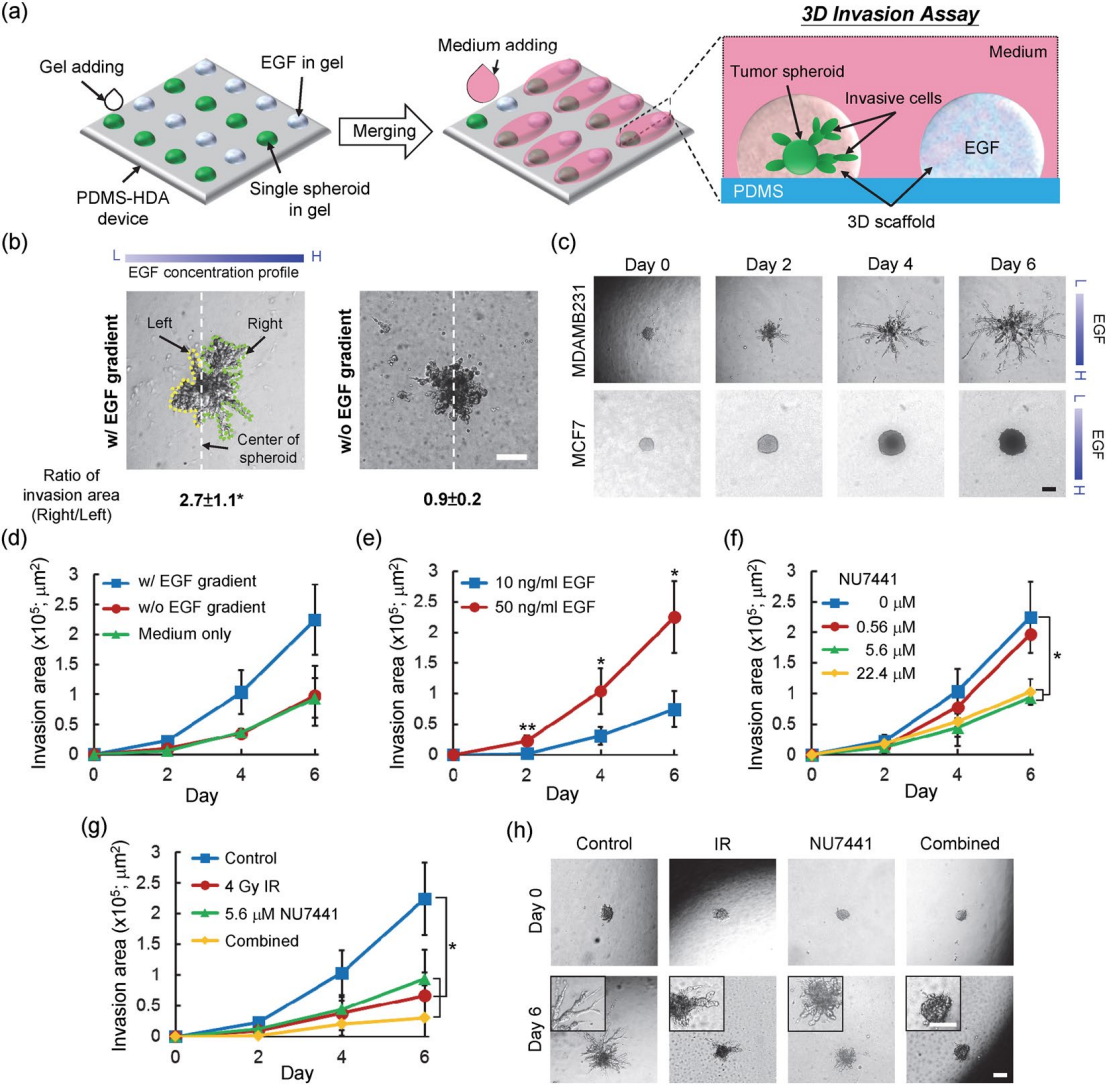
Three-dimensional cell invasion assay. (**a**) Design concept of the 3D invasion assay, combined with an EGF gradient generated *in situ* by PDMS-HDA. (**b**) Comparison of the oriented invasion of MDA-MB-231 spheroids with or without an EGF gradient over a 6-day culture. (**c**) Time sequence photographs showing the MDA-MB-231 (top panel) and MCF7 (bottom panel) spheroids during invasion with a 50 ng/ml EGF gradient. (**d**) Invasion area of MDA-MB-231 spheroids over a 6-day culture with either a 50 ng/ml EGF gradient, no EGF gradient (EGF presents in medium), or medium only. (**e**–**g**) Invasion area of MDA-MB-231 spheroids at different concentrations of EGF (**e**), different concentrations of NU7441 (**f**), and treatment with a combination of irradiation and drugs (**g**). Each data represents the mean ± SEM from 2 independent experiments (n = 4~6; *p < 0.05, **p < 0.01). (**h**) Corresponding morphology of MDA-MB-231 spheroid invasion at days 0 and 6 after treatment. Scale bars, 200 μm.

The PDMS-HDA platform could be adapted for investigating the invasive characteristics of tumor spheroids and specific biological attributes during cancer metastasis. An induced epithelial growth factor (EGF) gradient was incorporated into this device Fig. 5(a). Our results showed that the orientation of the invasive cells in relation to the spheroids was mediated and positively responsive to the EGF gradient Fig. 5(b). MDA-MB-231 spheroids exhibited spreading and tether-like features connecting the spheroid body and the invasive cells over the course of 6 days, whereas MCF7 spheroids had a tendency to proliferate Fig. 5(c). Under the stimulation of the EGF gradient, cells originated from MDA-MB-231 spheroids invaded into a larger area than that lacking the EGF gradient (EGF presented in medium) or cultured with no EGF Fig. 5(d). The invasive efficiency of the spheroids increased significantly with higher concentrations of EGF Fig. 5(e). In addition, the concentration of the collagen matrix dominated the invasive phenotype of the spheroid, in which cellular spheroids embedded in a higher concentration of collagen would result in a reduced invasion (Figure S6, Supplementary Information). Inhibition studies of MDA-MB-231 spheroid invasion by either NU7441 Fig. 5(f) or the combination of 5.6-μM NU7441 and 4-Gy irradiation Fig. 5(g) and (h) showed that the two doses of this drug (both 5.6 μM and 22.4 μM) could significantly increase the anti-invasion ability. Specifically, the combined treatment may further restrict tumor invasion of the spheroids.

Although the methodology of gradient generation was not optimized in this work, possibly because of the interference of external vibrations, it could be further improved by its integration with microfluidics, for example, to achieve a stable gradient^37, 38^. Taken together, our results indicate that the growth factor gradient generated on the device may contribute to spheroid invasion, providing a more appropriate *in vitro* cancer invasion model than the conventional Boyden chamber^30^.

Despite the advantages of our high-throughput approach, some limitations remain. First, the processing steps were conducted manually, requiring time and skill. To further confirm the reproducibility and reliability, an automatic robot system is needed to integrate the system into our current method, potentially meeting high-throughput and high-content drug screening requirements. Second, we adopted Matrigel as an *in vivo*-like scaffold material to support spheroid growth in 3D and immobilize them onto the device to facilitate medium replacement for long-term culture (longer than 1 week). Since cells cultured in different scaffold materials may respond differently from a physiological standpoint, they will need to be further studied in more detail.

Our findings using the PDMS-HDA approach showed that the synergistic effect of NU7441 and irradiation may improve the inhibition of *in vitro* tumor dissemination (Fig. 3) or invasion (Fig. 5). NU7441 is a potent inhibitor of DNA-PK kinase and induces persistence of DNA double-strand breaks (DSBs) and G2/M arrest in irradiated cells, increasing cellular sensitivity toward radiation and chemotherapy. In addition, NU7441 has been shown to restrict cancer progression and metastasis *in vivo* ^39, 40^, in accordance with the results of this work.

## Conclusions

We have presented a tumor spheroid culture platform performed by a PDMS-based hanging drop array and have integrated it with collagen fibrils to control microenvironmental behaviors and promote 3D modeling of tumor cells *in vitro*. Our results demonstrated that the collagen mixture could not only advance the interaction with cellular aggregates, but also boost the formation of tumor spheroids on PDMS surfaces within 24 h. This purpose-tailored microarray platform may be applied to tissue-based bioassays involving drug screening, tumor dissemination, cell co-culture, and tumor invasion. Events in the *in vivo*-like microenvironment could be examined by this platform too. Therefore, the present culture approach could be used as a versatile toolkit to study *in-vitro* tumorigenicity and cancer metastasis toward the development of personalized drug testing.

## Methods

### Cell lines

Human breast cancer cell lines MCF7 (HTB-22, ATCC) and MDA-MB-231 (HTB-26, ATCC) were maintained in α-minimum essential medium (α-MEM; SH30265, GE Healthcare), supplemented with 10% fetal bovine serum (FBS; S11150, Atlanta Biologicals) and 1% penicillin/streptomycin (P/S; 15140, GIBCO). The PC3 human prostate cancer cell line was maintained in RPMI–1640 medium (23400–021, GIBCO) supplemented with 10% FBS and 1% P/S. Head and neck squamous cell carcinoma (HNSCC) OSC19 and HN5 cells were maintained in Dulbecco’s modified Eagle medium (DMEM; SH30022FS, GE Healthcare), supplemented with 10% FBS, 1% P/S, 1 mM sodium pyruvate (11360070, GIBCO), 2 mM L-glutamine (25030081, GIBCO), 1% MEM vitamin (11120052, GIBCO), and 1% MEM non-essential amino acids (NEAA; 11140050, GIBCO). The HCT116 human colorectal carcinoma cell line and its derived cells – DNA-PKcs knock-out (KO) cells^41^ – were maintained in α-MEM supplemented with 10% FBS and 1% P/S. All cells were cultured in a humidified 5% CO_2_ incubator at 37 °C.

### Fabrication of PDMS-HDA device

The fabrication of the PDMS-HDA device relied on a flat substrate (i.e. 22*22 mm^2^ cover glass) fastened with an adhesive tape (3 M scotch 810), which is used as a mold to prevent leakage of the solution Fig. 1(a). Polydimethylsiloxane (PDMS; Sylgard 184, Dow Corning) mixture was then poured into the mold and cast at 125 °C for 1 h. The weight ratio of the base to the curing agent was 10:1, and the weight of the PDMS prepolymer was 0.5 g per chip. The chip was sterilized by UV for 30 min after removing the tape and stored in a sterilized dish by sealing with parafilm at room temperature until use.

### Three-dimensional spheroid culture by PDMS-HDA

Before the cells were loaded, a 1 ml type I collagen solution (~4.26 mg/ml; C3867, SIGMA) was prepared by adding cell culture medium and 1 N sodium hydroxide for pH adjustment (details are reported in the Supplementary Table S1). Before each individual experiment, the pH of the collagen solution was measured to be around pH 7.4. The cells were then resuspended in solutions with different concentrations of collagen (0, 50, 500, and 1000 μg/ml). In 3D multicellular spheroid culture Fig. 1(c), cellular drops with a volume of 1 μl were dispensed onto the PDMS-HDA device (step 1) by a liquid handing machine (Versa 10 spotter, Aurora Instruments Ltd.) Fig. 1(b). Each drop had an average diameter of 1.4 mm. The device was flipped and placed in a 6-cm cell culture dish, which had been pre-filled with medium of 750 μl and sealed with parafilm to prevent evaporation of the cell-containing drops. The whole set was transferred immediately to a humidified incubator at 37 °C overnight to allow the formation of collagen fibrils, which contributed to the sedimentation and aggregation of the cells (Steps 2 and 3), promoting the generation of cellular spheroids (Step 4).

### Evaluation of cell spheroid size

The mean volume of the 3D-cultured cell spheroids was calculated based on the measurements of their diameters by an open-source imaging software (Fiji). Although some spheroids were oval or shaped irregularly, the mean diameter (*l*) was determined by the following equation: *l* = (*a* × *b*)^1/2^, where *a* and *b* represent the two orthogonal diameters of each spheroid. The mean volume (*V*) of the spheroids was then evaluated by the equation $V=4\times \pi \times {(l/2)}^{3}/3$.

### Cell labeling and imaging

Cultures on the PDMS-HDA device were stained with a mixture of 2 μM calcein AM and 4 μM ethidium homodimer-1 (live/dead viability kit, L3224, Thermo Fisher Scientific Inc.) to stain for live and dead cells, respectively. The device was incubated under 5% CO_2_ at 37 °C for one hour. Bright field and fluorescent images were captured using a CCD camera (SPOT Flex, SPOT Imaging) on an upright microscope. Image analysis was conducted by the Fiji imaging software.

### Drug screening assay

To demonstrate that the PDMS-HAD device can screen for anti-cancer drugs, different tumor spheroids (of MCF7, MDA-MB-231, PC3, OSC19, and HN5 cells) were tested after treatment with the chemotherapeutic drugs paclitaxel (T7402, SIGMA) and cisplatin (P4394, SIGMA), at concentrations of 0 to 50 μg/ml. Based on the procedure described earlier, single cell spheroids were obtained at an initial cell density of 450/μl within each 500 μg/ml collagen-containing medium drop for a 2-day culture. Following the procedure shown in Fig. 2(a), a 1-μl drug-containing drop with two times the final testing concentration (0, 5, 25, or 50 μg/ml) was dispensed to the appropriate cell-containing drops, resulting in a total volume of 2 μl for each drop. After a 2-day drug treatment, cell viability was achieved using the CellTiter-Blue assay (at 520 nm excitation and 590 nm emission; G8080, Promega Corp.) with a volume of 1 μl per drop; cells were incubated for 4.5 h. A fluorescent microscope (BX51, OLYMPUS) was used to capture the fluorescent images of the cell-drug-containing drops. Fluorescent images were used to interpret the quantity of live cells. The fluorescent intensities of each drop were then analyzed by the Fiji imaging software. The relative cell viability, corresponding to the fluorescent intensity, was normalized against the untreated cells under different drug concentrations.

For *on-device* 2D drug screening, 1 μl of the cell suspensions mixed with 2 μg/ml collagen, used to facilitate the adhesion of the cells on PDMS, were first dispensed onto the device and cultured for 2 days without flipping. The following drug sensitivity experiments were conducted under the same conditions as the 3D drug screening mentioned earlier. For 2D control experiments from 96-well plates, cells were plated at a density of 6000 cells in 100 μl medium per well for 2 days. The media were exchanged accordingly with drug-containing media for an additional 2-day culture. Ten microliters of CellTiter-Blue were added to each well and the plates were incubated for 4.5 h. A plate reader (POLARstar Optima, BMG LABTECH) was used to determine the fluorescent intensity, and cellular viability was evaluated following the procedure described earlier.

### Tumor dissemination assay

The tumor dissemination assay was conducted following the procedure shown in Fig. 3(a). Tumor spheroids cultured for 2 days were generated from an initial cell seeding of 900 cells. The spheroid-containing drops were dispensed with 10 μl medium drops with or without the drugs. The resulting height of each drop (a total volume of 11 μl) was around 1.2 mm. For the combined treatment, drugs were added to the device before γ-ray irradiation. The device was flipped and placed on a 6-cm cell culture dish. The supported wall of the device had a height of 1.5 mm and sufficiently prevented the unwanted contact between the drop array and the examined substrate. The spheroid array was shifted to the substrate within seconds, followed by exertion with uniform pressure on the device, simultaneously separating it from the substrate see the example in Fig. 3(b). The volume of each shifted drop was around 5 μl. The dish was filled with the medium and sealed with parafilm to prevent evaporation. The dish was transferred to a 5% CO_2_ incubator at 37 °C overnight to allow the cell spheroids to adhere to the substrate. Medium with or without the drugs was added to the dish after 1 day. Cellular dissemination was observed daily under the microscope, and the migration area was derived by measuring cellular migration subtracted from the initial area at day 0.

### Three-dimensional co-culture assay

The PDMS-HDA device was adapted to the procedure shown in Fig. 4a to study whether two individual cell cultures in 3D could influence each other. The device is an emerging tool for modeling cell-cell interactions at different tissue levels^32^. Two cell spheroids (e.g. cell type A and type B) were generated at a gap of 1.5 mm. After culturing for 2 days, 1 μl of Matrigel was dispensed onto the cell-containing drops manually at 4 °C. Ten microliters of medium were added to the two adjacent drops after the gelation of Matrigel at 37 °C overnight. The mean diameters of two separate spheroids were observed daily. The spheroid volume was evaluated by the equation mentioned earlier, $V=4\times \pi \times {(l/2)}^{3}/3$.

### Three-dimensional invasion assay

To perform the tumor spheroid invasion assay with the PDMS-HDA device, cell spheroids were first generated on the device for 24 h, following the procedure shown in Fig. 5(a). Three-dimensional scaffold matrices of 10 μl were added to each cell spheroid-containing drop, which was mixed with Matrigel and collagen-I (in PBS at pH 7.4) at a ratio of 1:1. The final concentration of Matrigel was 5 mg/ml and the concentration of collagen-I was 1.5 mg/ml. The addition of collagen-I to Matrigel facilitated the invasion by increasing matrix stiffness^42^. We observed that the cells in the mixed gel invaded more effectively than Matrigel or collagen gel used alone (data not shown). Five microliters of epidermal growth factor (EGF; 236-EG-200, R&D systems)-encapsulated scaffold matrices were dispensed beside the corresponding spheroid drop at a gap of 1.5 mm. The EGF was used at concentrations of 0, 10, and 50 ng/ml. After gelation of the scaffold matrix at 37 °C for 1 h, 20 μl of standard culture media or media containing NU7441 were dispensed and connected with the two adjacent gel drops at day 0. The addition of the medium allows the generation of an *in vitro*-like microenvironment, in which a gradient of EGF from the encapsulated matrix could be generated to stimulate and coordinate nearby spheroid invasion Fig. 5(a). For the combination treatment, drugs were added to the device before γ-ray irradiation. Spheroid invasion was observed daily by microscopy. In addition, the invasion area was evaluated as a measurement of cellular spreading subtracted by the initial area at day 0.

### Statistical analysis

Student’s t test was used to compare data from two groups. The one-way TukeyHSD ANOVA test was used to compare data from more than two groups. A p < 0.05 was considered to be statistically significant.

## Electronic supplementary material

Supplementary information
