# Supplementary material for: Three-dimensional spheroid culture targeting versatile tissue bioassays using a PDMS-based hanging drop array
Source: Sci Rep. 2017 Jun 29;7:4363. doi: 10.1038/s41598-017-04718-1 (PMC5491519; doi:10.1038/s41598-017-04718-1)
Supplement: Supplementary file 1 — Supplementary information [file 41598_2017_4718_MOESM1_ESM.doc]

Supplementary Information for

**Three-dimensional spheroid culture targeting versatile tissue bioassays using a PDMS-based hanging drop array**

Ching-Te Kuo^1,2^, Jong-Yueh Wang^2^, Yu-Fen Lin^1^, Andrew M. Wo^3^,

Benjamin P. C. Chen^1*^, and Hsinyu Lee^2*^

*^1^Division of Molecular Radiation Biology, Department of Radiation Oncology,*

*University of Texas Southwestern Medical Center, Dallas, TX, USA*

*^2^Department of Life Science, National Taiwan University, Taipei, Taiwan, R.O.C.*

*^3^Institute of Applied Mechanics, National Taiwan University, Taipei, Taiwan, R.O.C.*

*^*^ Prof. Benjamin P. C. Chen. E-mail: benjamin.chen@utsouthwestern.edu or*

*^*^ Prof. Hsinyu Lee. E-mail: hsinyu@ntu.edu.tw*

**Supplementary information file includes 6 figures, 1 table, and 1 reference.**

Supplementary Table

**Table S1. Preparation of collagen-I mixture**

| **Collagen concentration (μg/ml)**  **Content of mixture (total 1 ml)** | 0 | 50 | 500 | 1000 |
| --- | --- | --- | --- | --- |
| Cell culture medium (μl) | 1000 | 987.9 | 879.8 | 759.6 |
| Collagen-I (4.26 mg/ml; μl) | 0 | 11.8 | 117.5 | 235 |
| NaOH (1 N; μl) | 0 | 0.3 | 2.7 | 5.4 |

Supplementary Figures


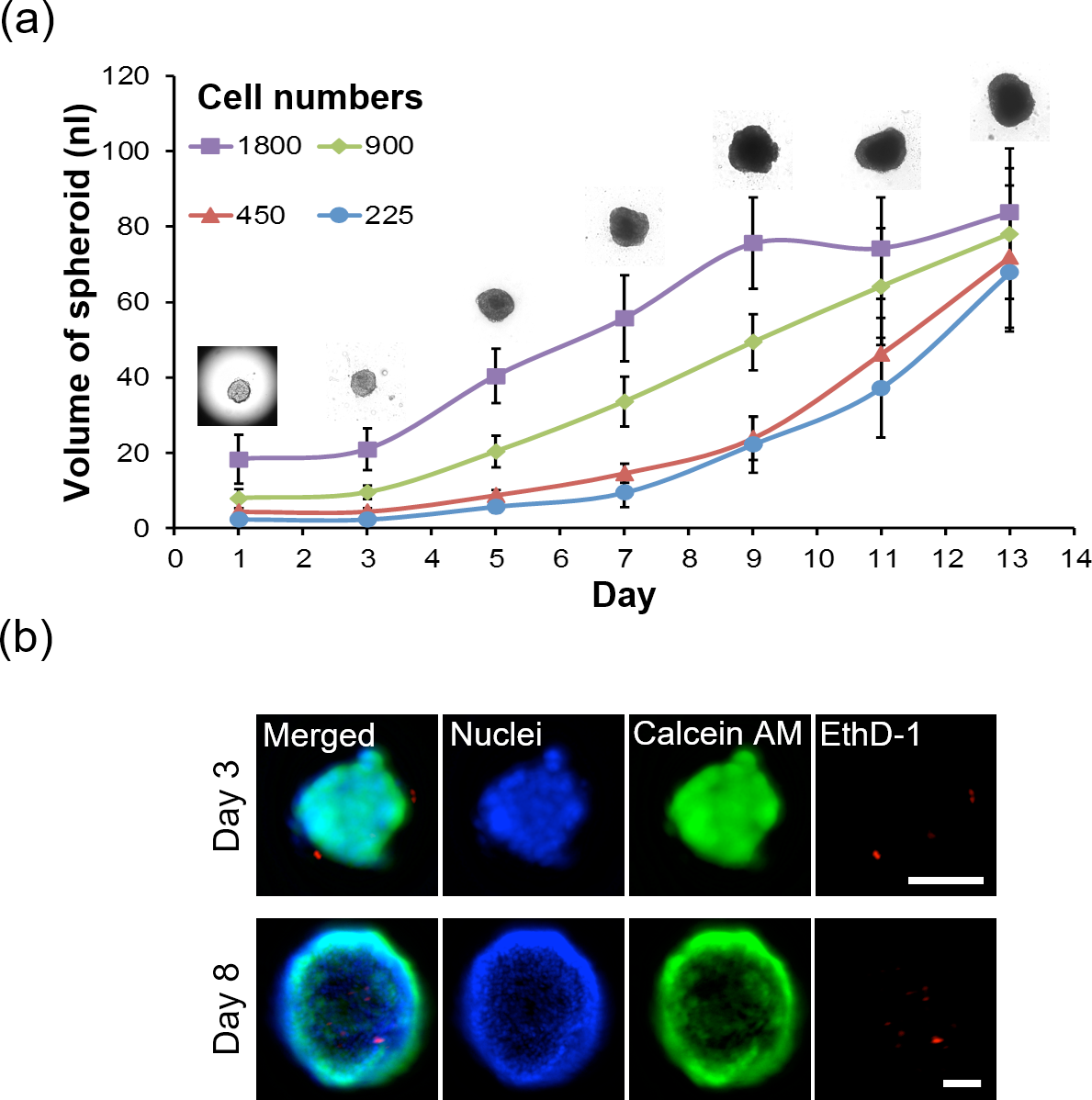


**Figure S1. Long-term culture of MCF7 spheroids by PDMS-HDA.** (a) Different numbers of MCF7 cells were applied to 3D spheroid cultures. Volumes of MCF7 spheroids over 13 days were measured. To facilitate the medium change properly, we filled a 1 μl basement membrane matrix (Matrigel with a final concentration of 5 mg/ml at 4 °C; 354234, Corning) on each spheroid drop and immediately incubated them at 37 °C overnight. The adoption of Matrigel was to immobilize the spheroid drops accordingly to their initial locations during medium exchanges. Afterwards, cell culture medium was filled over the device. Medium was changed every 2 ~ 3 days to sustain the nutrients and osmolality suitable for cells. The data are presented as mean ± SD (n = 8 ~ 13 for each initial cell number condition). (b) MCF7 spheroids were stained with live cells (Calcein AM; green), dead cells (EthD-1; red), and nuclei (Hoechst; blue) on days 3 and 8. Scale bars, 100 μm.


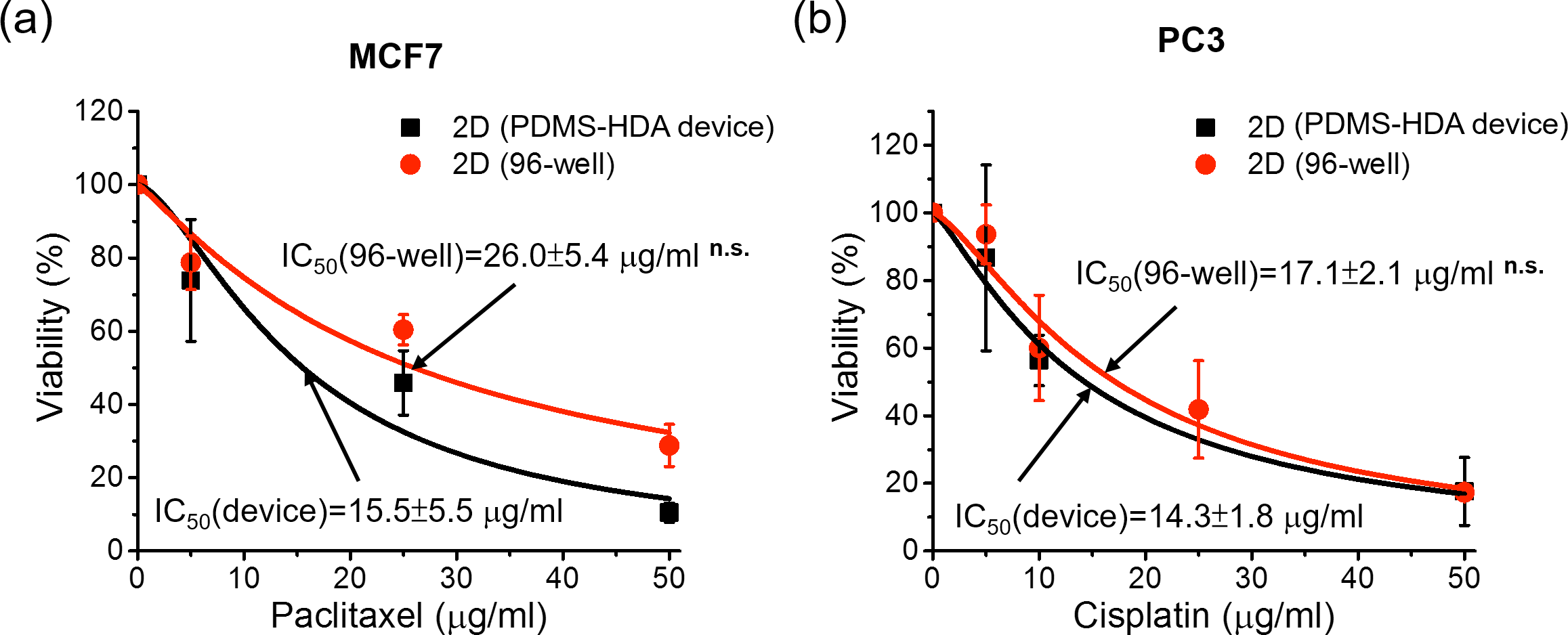


**Figure S2. Comparison of toxicity profiles derived from 96-well plates and PDMS-HDA devices.** (a) Viability of MCF7 cells treated with paclitaxel for 48 h. (b) Viability of PC3 cells treated with cisplatin for 24 h. Cell viability with different drug concentrations was normalized against untreated cells. No statistical significance was found in the dose response between 96-well plate and PDMS-HDA device. The IC_50_ values were fitted by a four-parameter logistic equation presented[^1^](#_ENREF_1). Data are presented as mean ± SEM from 2 experiments (n = 8 ~ 10) in (a) and data as mean ± SD from n = 5 in (b).


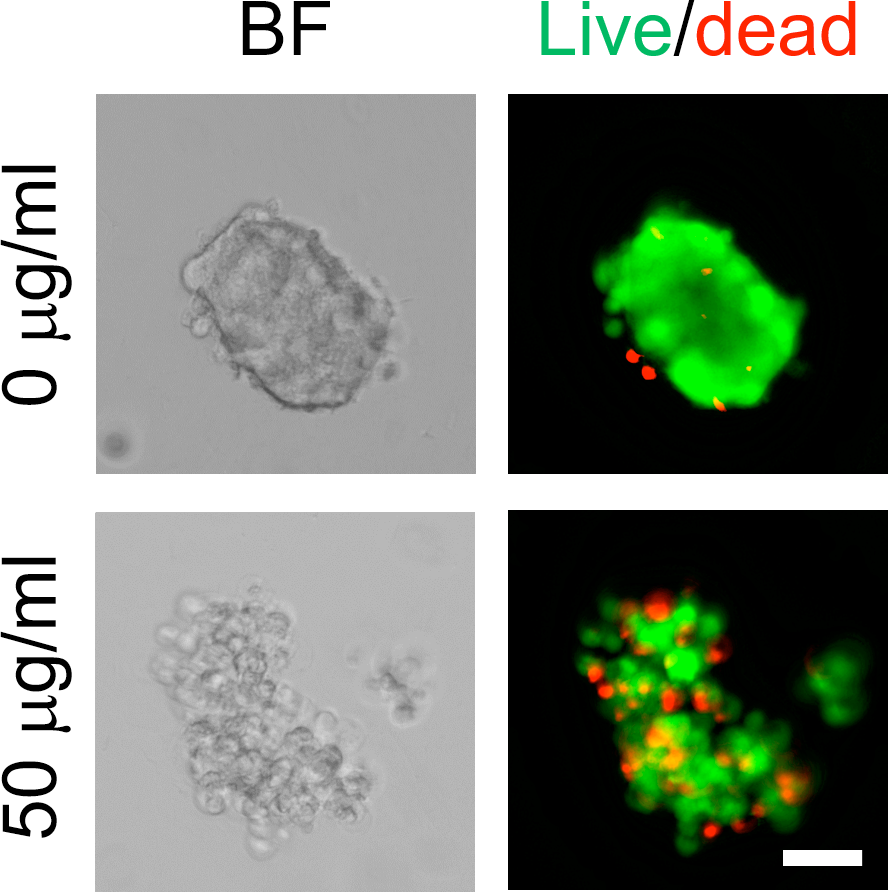


**Figure S3. Live and dead cells staining of MCF7 spheroids after the 24-h paclitaxel treatment.** Scale bar, 100 μm.


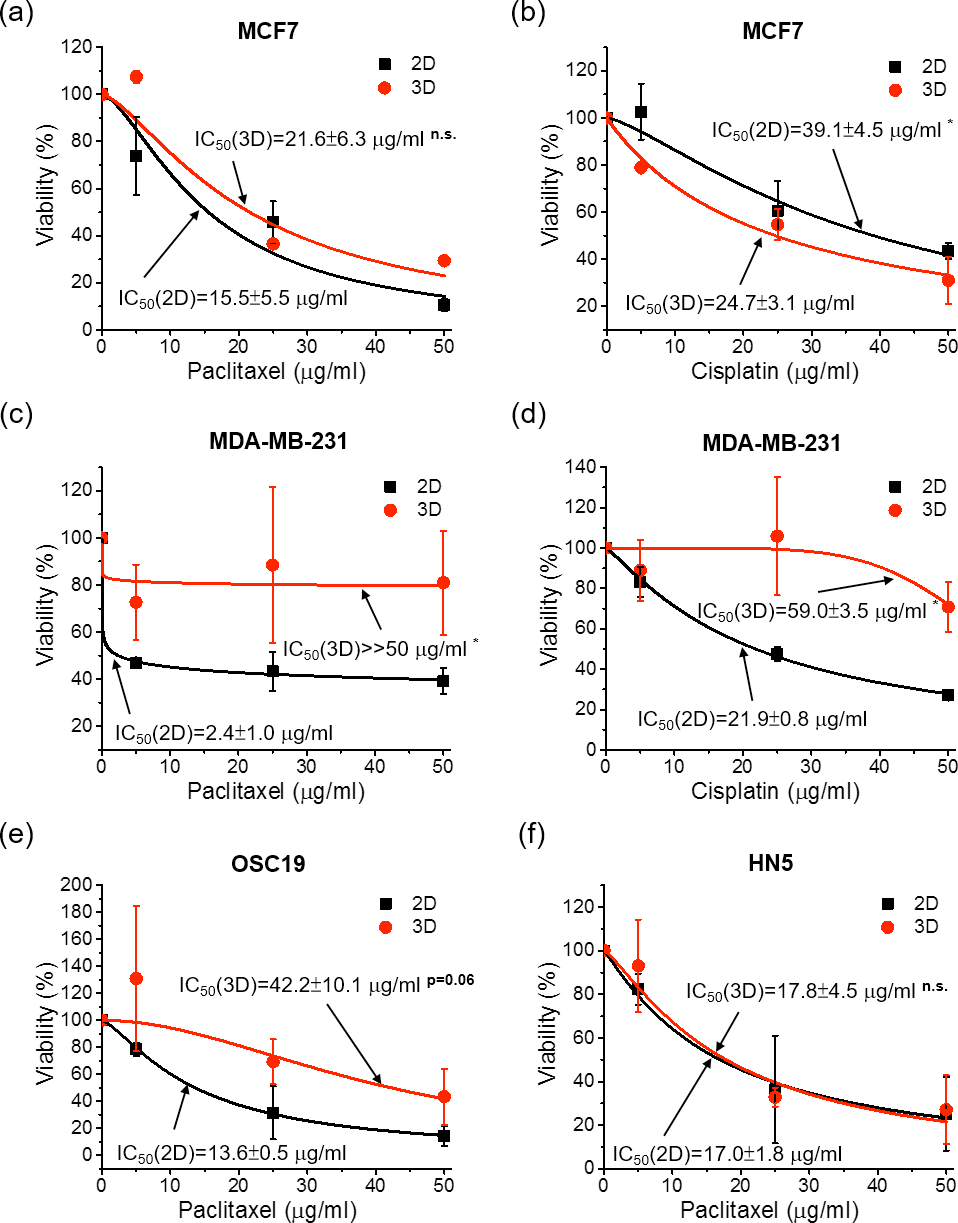


**Figure S4. Comparison of toxicity profiles between 2D and 3D cultured cells.** Toxicity profiles of paclitaxel or cisplatin were determined in MCF7 [(a) and (b)], MDA-MB-231 [(c) and (d)], OSC19 (e), and HN5 cells (f), cultured in 2D or 3D for 48 h. Cell viabilities with different drug concentrations were normalized against that of untreated cells. Results showed that 3D cultured MDA-MB-231 cells are more resistant than 2D cultures, for the two commonly-used anti-cancer drugs [(c) and (d)]. However, no significant difference was observed among the IC_50_ values obtained from 2D and 3D cultures (i.e. MCF7, OSC19, and HN5), although the mean IC_50_ (42.2 μg/ml) of 3D OSC19 was higher than 13.6 μg/ml from 2D (p = 0.06) [(a), (e), and (f)]. Note that, the 2D-cultured MCF7 cells was more cisplatin resistant than 3D culture (b). The IC_50_ values fitted by a four-parameter logistic equation were shown[^1^](#_ENREF_1). Each data represents the mean ± SEM from 2 ~ 3 independent experiments. *, p < 0.05.


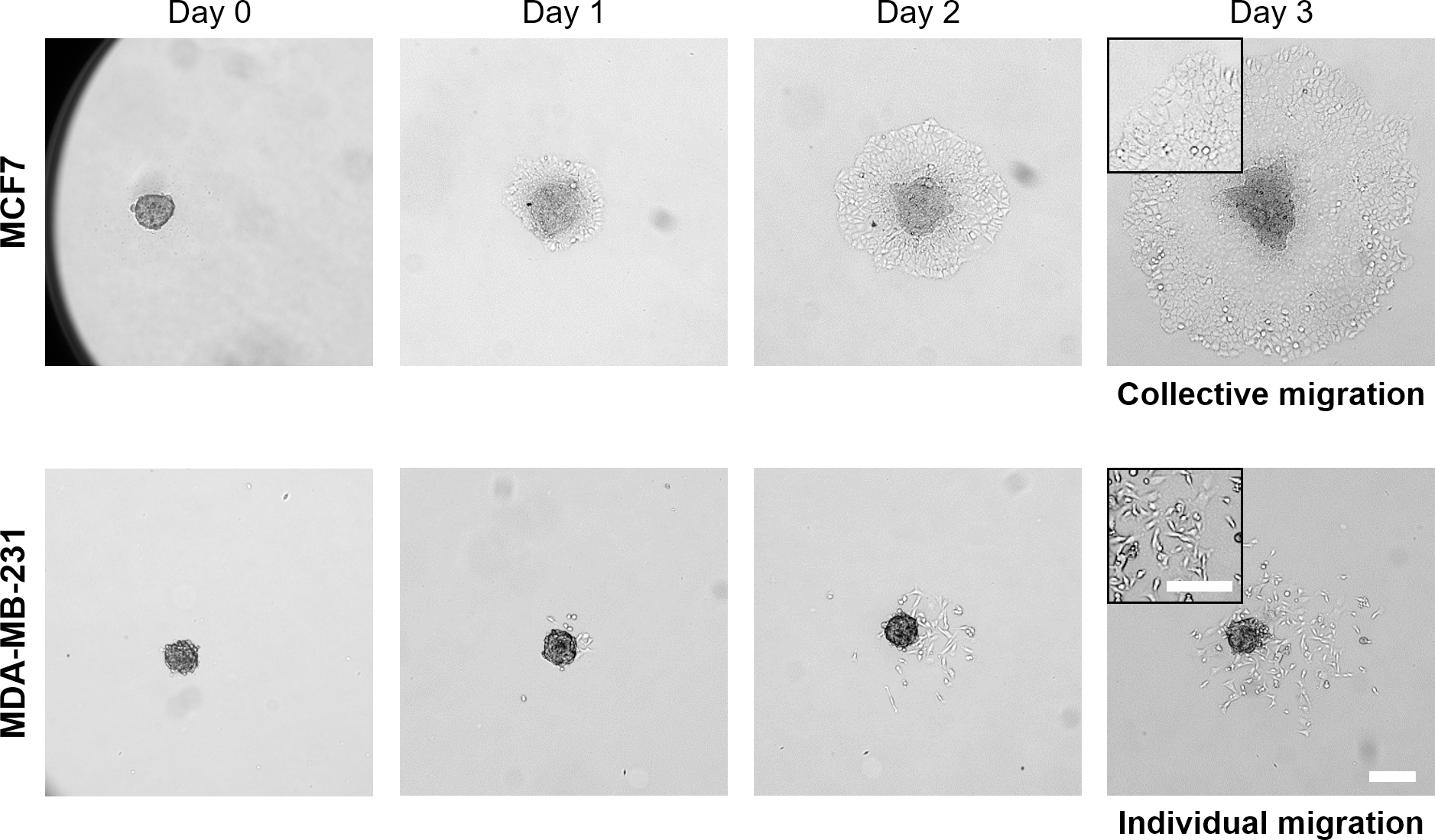


**Figure S5. Comparison of spheroid dissemination patterns between MCF7 and MDA-MB-231 cells.** A collective migration pattern was observed in MCF7 spheroids under dissemination assay, whereas MDA-MB-231 spheroids displayed in an individual and discrete migration pattern. Note that the migratory cell numbers from the MDA-MB-231 spheroids were less than the MCF7 spheroids. Scale bars, 200 μm.


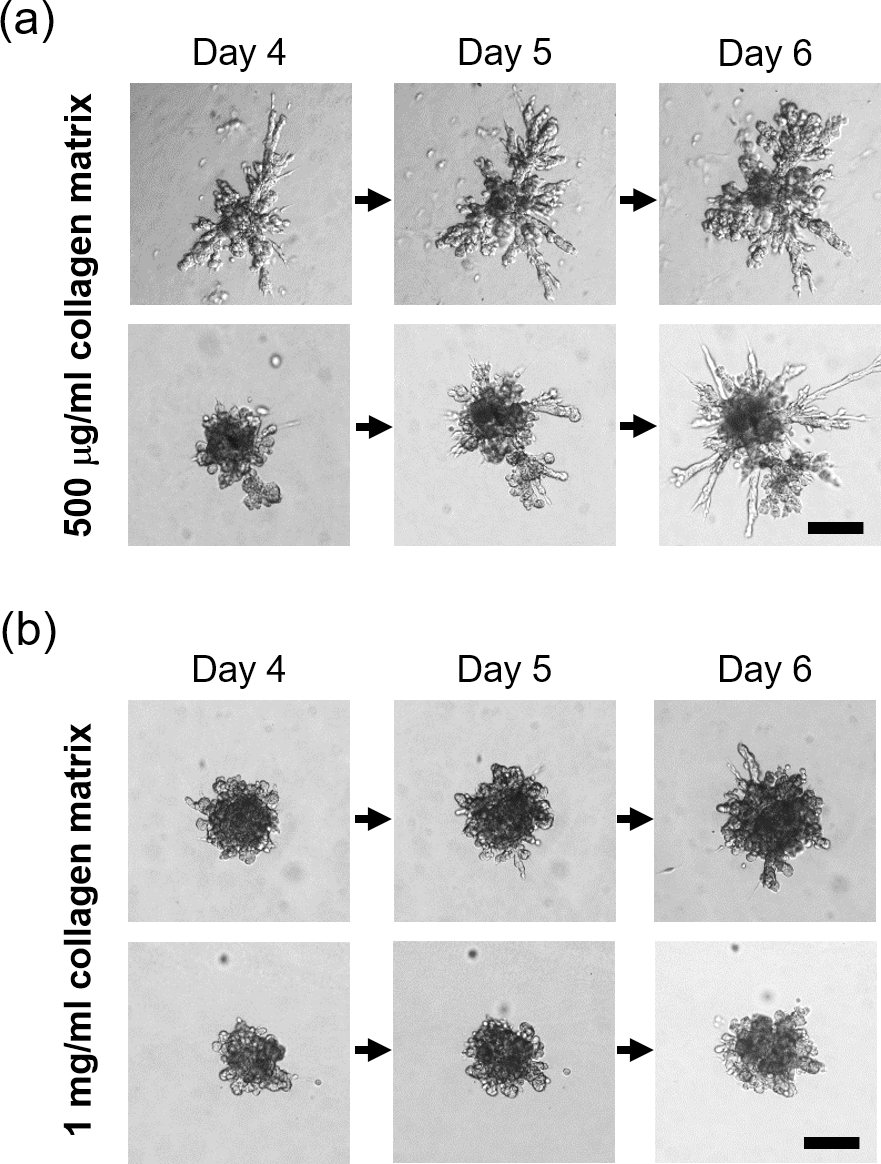


**Figure S6. Effect of collagen matrix on the tumor spheroid invasion.** MDA-MB-231 spheroids were applied to spheroid invasion assay under 500 μg/ml (a) or 1 mg/ml (b) collagen matrix in the presence of EGF (50 ng/ml) gradient. Two representative spheroids in each condition were shown at day 4 to 6. Results showed that a limited invasion efficiency is observed in the presence of 1mg/ml collagen matrix, subject to a smaller pore size and a higher stiffness than the 500 μg/ml collagen used. It implies that the physical constraint in cellular scaffolds may dominate the tumor invasion. Scale bars, 200 μm.

**Reference**

1. Hsiung, L. C. et al. Dielectrophoresis-based cellular microarray chip for anticancer drug screening in perfusion microenvironments*. Lab Ch*i**p 11**, 2333-2342 (2011).
